# Gastroenterologically relevant high alert medications prescribed to children with chronic diseases—a consensus-driven single-center pilot study

**DOI:** 10.3389/fped.2026.1812750

**Published:** 2026-05-04

**Authors:** Judith Hochrainer, Rebecca Einspieler, Andreas Heilos, Judith Pichler, Michael Boehm

**Affiliations:** Division of Pediatric Nephrology and Gastroenterology, Department of Pediatrics and Adolescent Medicine, Comprehensive Center for Pediatrics, Medical University of Vienna, Vienna, Austria

**Keywords:** chronic disease, gastroenterology, home environment, medication error, patient safety, pediatric, risk management

## Abstract

**Introduction:**

Children with chronic diseases, including complex gastroenterological patients, often manage complex home medication regimens, where errors pose significant patient- and medication-related risks. High Alert Medications (HAMs) are those with a particularly high potential for adverse consequences if administered incorrectly.

**Aim:**

This study used a consensus approach to identify HAMs in the pediatric gastroenterological population, using a new scale to measure medication risk and potential patient harms.

**Methods:**

This secondary analysis included 106 children with chronic diseases discharged from the Medical University of Vienna. Interdisciplinary teams identified relevant medications through a consensus approach and separately evaluated the inherent risk of each medication and the potential harm of its use at the individual patient level using five-level scales. Substances were coded via the Anatomical Therapeutic Chemical classification system (ATC system), ranked, and compared with existing HAM lists.

**Results:**

Thirty-two medications were categorized into the highest medication risk categories, and 16 into the highest potential patient harm group. The intersection of these categories identified 12 medications defined as HAMs, with immunosuppressants forming the largest therapeutic class. Notably, 50% of the identified HAMs appeared in only one other published list, and one-third were not previously classified as High Alert.

**Discussion:**

Immunosuppressants carry elevated risk due to narrow therapeutic windows, complex drug interactions, and potential for severe organ dysfunction. Discrepancies between HAM lists stem from varied methodologies and local clinical characteristics. This study distinguishes itself by focusing specifically on home care and by including off-label medications. The findings underscore the need for localized HAM lists to address specific risks within the target population. Validation studies and consideration of empirical data are needed to further generalize our findings.

## 1 Introduction

Children suffering from severe chronic diseases, internationally recognized as Children with Medical Complexity, represent a particularly vulnerable group characterized by chronic conditions, functional impairments, special needs, and high healthcare utilization (1). This population also includes those requiring special pediatric gastroenterological long-term care (2). In recent years, there has been an increase in the number of children suffering from chronic illnesses because of our high standard of care and rising survival rates (3). These patients and their caregivers must frequently manage complex medication regimens at home. Errors result in risks to patient safety. Factors that influence the potential for harm from medication errors can be categorized in patient- and medication-related (4).

Medications that are classified as high-alert are characterized by a particularly high potential for adverse consequences if administered incorrectly (5). To prevent adverse drug events, organizations have developed critical medication safety frameworks. For example, the Institute for Safe Medication Practices (ISMP) maintains guidelines on High-Alert Medications (HAMs) (6), and the Pediatric Pharmacy Association (PPA) established the “Key Potentially Inappropriate Drugs in Pediatrics” (KIDs List) to identify potentially inappropriate and high-risk drugs in pediatrics (7). Several publications have defined lists of such high-alert medications, however, only few of them are related to the pediatric population, and none for pediatric patients with chronic diseases who take their medications at home.

In a recent study, we found medication-misunderstandings in 80% of home-treatments in children with chronic diseases, predominantly in families with communication barriers (due to migratory background) and with newly prescribed medications (4). Despite the need for complex pharmacological regimens in this population, there are currently no established estimates of medication-related problems among pediatric gastroenterology patients managed in the home environment.

In the present study we performed a secondary analysis of this data to define high-alert medications with gastroenterological relevance prescribed for home-treatment in pediatrics.

## 2 Methods

The source data was prospectively collected in 106 children with chronic diseases who required home pharmacotherapy and were discharged between May 2018 and January 2019 from the Division of Pediatric Nephrology and Gastroenterology at the Medical University of Vienna, a tertiary pediatric center (4). Data on 639 drug prescriptions were extracted from electronic health records and discharge letters (4). The original study′s objective was to assess risk factors for potential harm at the patient level with no assessment of individual prescribed medication.

Here, we assessed risk factors at the medication level (= identification of high alert medications) with relevance in pediatric gastrointestinal indications by a *post-hoc* analysis of the source data:

First, a consensus-based approach was used to identify the medications relevant to pediatric gastroenterology. Two clinical specialists independently reviewed the original medication list, annotating both disease-specific agents and broader therapeutic classes commonly used in pediatric gastroenterology. Discrepancies were resolved through a structured consensus discussion moderated by a third senior clinician, ensuring that the final list reflected clinical practice.

**Figure 1:**
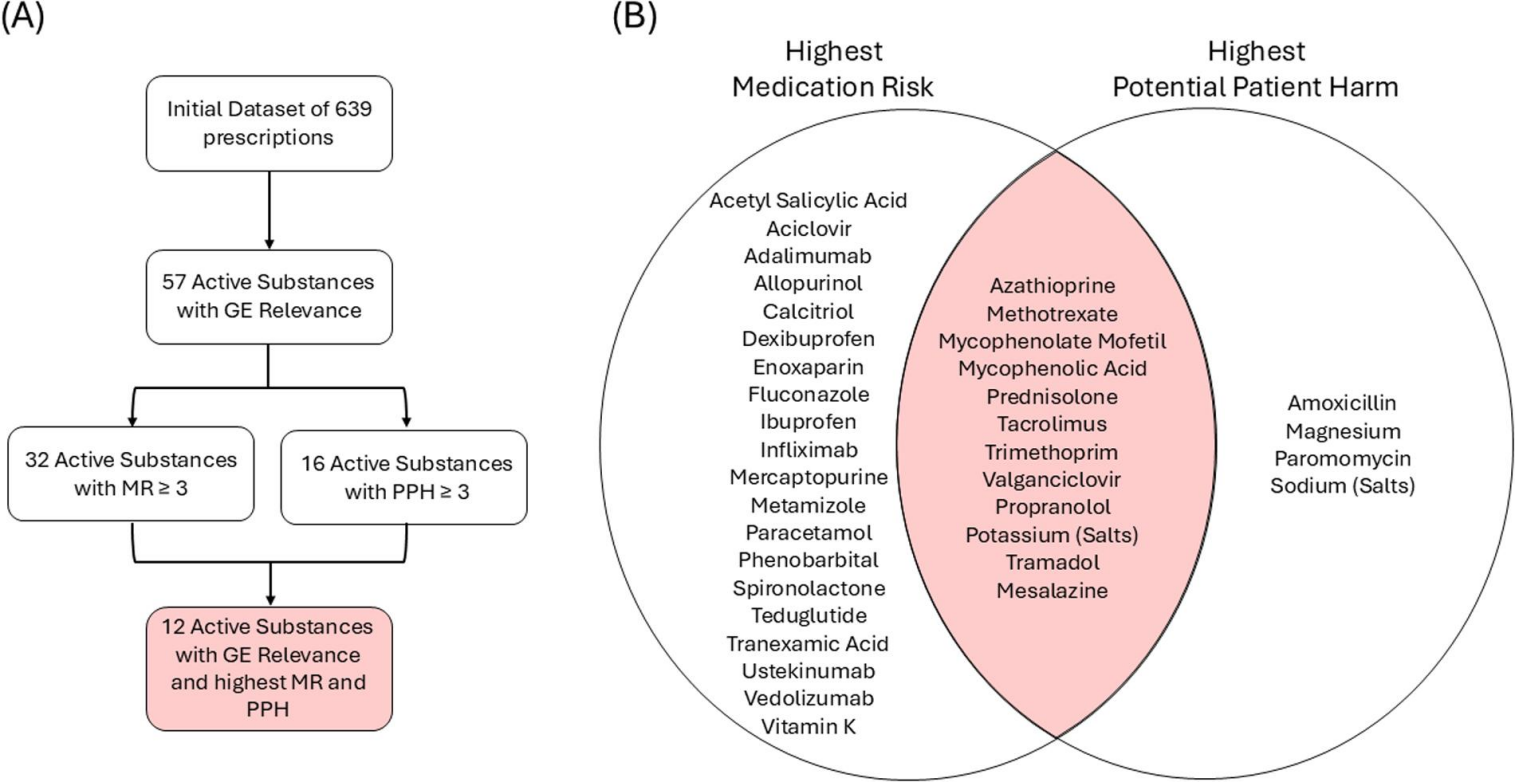
**(A)** Flow diagram depicting the systematic filtering process to identify potential high-alert medications in pediatric gastroenterology out of 57 active substances **(B)** different active substances (*n* = 36) with a “serious to severe” risk (score ≥ 3) in at least one of two categories: potential patient harm (PPH) or medication risk (MR). The screening resulted in 36 unique substances.

As shown in Figure 1A, 57 active substances with gastroenterological relevance were identified from the initial set of medications, prescribed to 91 patients, predominantly female (*n* = 55, 60.4%), with a median age of 9.6 years (Interquartile Range: 3.8–13.9). This cohort included 42 patients (46.15%) with a primary gastroenterological diagnosis.

The conceptual framework guiding our approach to identify HAMs is based on dual perspective risk assessment tailored to the pediatric home-care environment. Therefore, we categorize medication safety not only by the intrinsic pharmacological danger of a drug (Medication Risk, MR) but also by adding patient-specific characteristics (Potential Patient Harm, PPH). The intersection of high MR and high PPH defines a relevant High Alert Medication at the level of a specific population.

For each medication, MR and PPH were separately assessed by two independent interdisciplinary teams using a five-level scale adapted from the HAMEC score by Gates et al. (8) Both, MR and PPH were each rated from ‘no harm’, ‘minor harm’, ‘moderate harm’ to ’serious harm’ and ’severe harm’ To quantify these assessments, the categories were assigned numerical values ranging from 0 to 4, with 0 indicating ‘no harm’ to 4 ’severe harm’.

First, MR was categorized for each medication by two pediatricians and two pharmacists. Subsequently, PPH was categorized for each medication by a pediatrician and a pharmacist at the patient-level by integrating MR ratings with information from the patientś discharge letter, using the highest PPH for analysis. Assessments were individually performed and disagreements of >1 category were resolved through discussion, when needed with an additional team member, until a consensus was reached (4).

All substances were coded according to the Anatomical Therapeutic Chemical classification system (ATC system) to ensure standardized reporting and ranked according to their MR and PPH levels. This HAM list was compared with previously published HAM lists identified through a literature search (Pubmed) and their reference lists.

Each medication was checked for its presence in these external references, either as a specific agent or as part of a broader therapeutic class. This allowed for categorization of medications based on concordance with external standards.

Statistical Analysis: Descriptive statistics were used to summarize patient demographics and medication characteristics. The positive predictive value was calculated to quantify the overlap between the study's HAM list and established external lists. All statistical analyses were performed using IBM SPSS Statistics for Windows, Version 30.0.0.0 and R version 4.5.2.

### Ethics statement

Study protocol was approved by the local institutional ethics committee (EK 1351/2015). All data were anonymized prior to analysis to protect patient confidentiality and privacy.

## 3 Results

As shown in Figure 1A, 32 medications (almost 60%) were categorized to the highest MR categories. MR was classified as serious in 42.1% and severe in 14.0% of cases. The highest PPH group comprised 16 medications. The integration of specific patient data for PPH therefore led to a significant consolidation in risk assessment with a reduction to nearly 30% of the medications (17.5% serious, 10.5% severe). The most critical group, found at the intersection of the highest categories of both MR and PPH, included a core list of 12 medications that are defined as high-alert medications by our approach (Figure 1, Table 1).

**Table 1:** High alert medications sorted by medication category with occurrence in 8 published pediatric HAM lists.

Active Substance	ATC code; Medication Name(s)	HAM List (see References)								approved in pediatrics
		11^a^	10^a^	9^c^	12^b^	13^b^	14^b^	5^b^	7^c^	
**Immunosuppressants**										
Azathioprine	L04AX01 Imurek, Azathioprin			X						yes
Methotrexate	L04AX03 Methotrexat, Ebetrexat, Metoject	X		X		X				yes
Mycophenolate Mofetil	L04AA06 Cellcept			X						yes
Mycophenolic Acid	L04AA06 Myfortic			X						no
Prednisolone	H02AB06 Aprednislon, Prednisolut			X					X	yes
Tacrolimus	L04AD02 Prograf, Modigraf		X	X						yes
**Antibiotics/Antivirals**										
Trimethoprim	J01EE01 Eusaprim									yes
Valganciclovir	J05AB14 Valcyte, Valganciclovir									yes
**Antihypertensive Drugs**										
Propranolol	C07AA05 Inderal, Propranolol									yes
**Elektrolytes**										
Potassium (Salts)	B05XA01 Kal Chlor A12BA30 Kalioral		X	X	X	X	X	X		yes, no
**Opioid Analgesics**										
Tramadol	N02AX02 Noax, Tramal					X			X	yes
**Antiinflammatory**										
Mesalazine	A07EC02 Pentasa, Pentasa Ret, Salofalk, Claversal, Mesagran								X	yes

a Expert based HAM determination.

b Clinical Evidence based HAM determination.

c combined HAM determination.

The largest therapeutic class were immunosuppressants (Azathioprine, Methotrexate, Mycophenolate Mofetil, Mycophenolic Acid, Prednisolone, Tacrolimus).

As shown in the Table 1, we benchmarked the high-alert medications from our core list, defined as those with an overlap of high MR and high PPH, against eight published pediatric HAM lists (5, 7, 9–14). Our HAM list showed a positive predictive value of 0.67: Of all the medications classified as HAM in our list, two-thirds had been previously classified as high-alert in at least one other list. However, the degree of overlap varied significantly: every second HAM was found only in a single list (e.g., Azathioprine, Mycophenolate Mofetil, Mycophenolic Acid, Prednisolone, Tramadol). One-third appear only on our list and are missing from the other lists (e.g., Trimethoprim, Valganciclovir, Propranolol, Mesalazine).

## 4 Discussion

The results are likely descriptive of the needs of a population treated at a tertiary care setting. While MR refers to the generic risk associated with a given medication, PPH integrates relevant clinical information associated with the patients and their diseases.

Immunosuppressants as our largest therapeutic class are consistent with recent literature, which confirms immunosuppressants as high-alert medications (9), even though established HAM lists like ISMP or the KIDs List of the Pediatric Pharmacy Association do not explicitly list them as high-alert.The heightened risk for adverse events stems from their narrow therapeutic window, high potential for complex drug-drug interactions [e.g., Tacrolimus and Cyclosporine (10)], and severe adverse effects such as organ dysfunction (15) [e.g., Tacrolimus with nephrotoxic and gastrointestinal adverse events (10)] and electrolyte imbalances (15). Their complex dosing, particularly in pediatric patients, and the complexity of thorough monitoring further contribute to a high risk of errors with devastating consequences.

The divergencies between our list and the other published ones are likely rooted in the different areas and methodologies. Four of the seven studies are over 10 years old, likely covering different clinical settings. Some lists are based purely on expert opinion—a method similar to our MR estimations (10, 11). Others are based on incidence reports, which, like our PPH data, introduce information at the clinical level (5, 12–14). The study of Maaskant et al. combined both techniques to a hybrid approach that might explain the good overlap with our findings; for instance, Maaskant et al. were the only to identify ‘Immunosuppressive drugs’ as a high-risk class (achieving ≥75% expert consensus), which strongly supports our specific inclusion of multiple drugs from this category (9).

Accordingly, our analysis revealed a moderate overlap between our generated list and the established international frameworks, resulting in a relatively low positive predictive value. This finding, however, does likely not represent a methodological error, but rather demonstrates that approximately one third of HAMs prescribed to pediatric patients for home treatment are currently absent from standard HAM lists. Two critical contextual factors differentiate our study from all these lists: we focused exclusively on gastroenterology relevant medications used in a home-care setting, whereas comparator lists often include medications administered in acute hospital settings (e.g., intravenous drugs). Our focus on the outpatient setting reveals a critical safety gap when compared to acute care. Another study showed that hemodynamic alterations and electrolyte disturbances are consequences of HAM interactions in the PICU. They recommend close clinical monitoring for these patients (16). In the home-care setting, however, this approach is not feasible. This underscores the necessity of HAMs in outpatient use to further develop targeted education rather than clinical monitoring. Furthermore, our list includes medications used off-label in our division (e.g., Myfortic), which are unlikely to appear on broader, consensus-based lists.

Recently, in the study by Selzer et al. (4), we demonstrated the influence of population characteristics on medication understanding and patient safety. While the identified patient-related factors, such as the socio-cultural and economic composition of the patient population, have no influence on patient-independent MR, they certainly do influence PPH. Depending on the local prevalence of groups with a migratory background who face communication barriers, the HAM list will therefore vary from center to center despite similar indications and medications. A medication that is disproportionately prescribed to patients with migratory background may have a disproportionately higher PPH – despite a similar MR – than a medication that is more commonly used in low-risk populations defined in our previous work (4).

Our approach corresponds to that challenge and follows the recommendations for adapting HAM lists to the local situation (9). In our case, the frequency of immunosuppressants as HAM reflects the typical distribution of diagnostic categories in the given population at our tertiary care center. This population includes transplant recipients as well as patients with autoimmune diseases (e.g., inflammatory bowel disease, autoimmune hepatitis, etc.) with a high prevalence of families from a migratory background [54.7% of primary caregivers (4)]. Importantly, specific measures for these few selected HAMs can have an exponentially positive influence on patient safety, particularly when tailored for high-risk populations. Center specific lists of HAM can thus guide the effective development and implementation of interventions to increase patient safety in children with chronic diseases. Our study suggests the HAM evaluation process combining MR and PPH as a robust and feasible tool that allows to select a dozen HAMs from several hundred prescriptions, for which targeted measures can then be implemented to improve patient safety. Validation studies in comparable independent populations and consideration of empirical data on medication errors and adverse effects are needed to further generalize our findings.

## Data Availability

The data analyzed in this study is subject to the following licenses/restrictions: The data set is only available upon justified request. Requests to access these datasets should be directed to Dr Michael Boehm, michael.boehm@meduniwien.ac.at.
